# Impact of *CYP2E1*, *GSTA1* and *GSTP1* gene variants on serum alpha glutathione S-transferase level in patients undergoing anaesthesia

**DOI:** 10.1186/s12881-016-0302-6

**Published:** 2016-05-14

**Authors:** Adam Mikstacki, Marzena Skrzypczak-Zielinska, Oliwia Zakerska-Banaszak, Barbara Tamowicz, Maria Skibinska, Marta Molinska-Glura, Marlena Szalata, Ryszard Slomski

**Affiliations:** Department of Anaesthesiology and Intensive Therapy, Regional Hospital, Juraszow 7/19, 60-479 Poznan, Poland; Institute of Human Genetics, Polish Academy of Sciences, Strzeszynska 32, 60-479 Poznan, Poland; The NanoBioMedical Centre, Adam Mickiewicz University, Umultowska 85, 61-614 Poznan, Poland; Department of Genetics in Psychiatry, University of Medical Sciences, Szpitalna 27/33, 60-572 Poznan, Poland; Department of Computer Science and Statistics, University of Medical Sciences, Dabrowskiego 79, 60-529 Poznan, Poland; Department of Biochemistry and Biotechnology, University of Life Sciences, Dojazd 11, 60-632 Poznan, Poland

**Keywords:** *GSTA1*, *GSTP1*, *CYP2E1*, Polymorphism, α-GST, Hepatotoxicity, Sevoflurane

## Abstract

**Background:**

The serum glutathione S-transferase alpha (α-GST) concentration has been used as a marker of hepatic condition. After sevoflurane anaesthesia a mild impairment of hepatocellular integrity was observed. Genetic polymorphisms in *CYP2E1*, *GSTA1* and *GSTP1* genes, affecting enzymes activity, may possibly influence the hepatotoxic effect of sevoflurane. The aim of this study was to assess the influence of genetic polymorphism of *CYP2E1*, *GSTA1* and *GSTP1* genes on serum α-GST level in 86 unrelated patients representing ASA physical status I-II, undergoing laryngological surgery under general anaesthesia with sevoflurane.

**Methods:**

The serum samples from three perioperative time points were analyzed using ELISA. Genetic variants were detected by pyrosequencing and sequencing. Finally, the statistical associations between serum α-GST concentration and analyzed alleles of *CYP2E1*, *GSTP1* and *GSTA1* genes were estimated.

**Results:**

The allele GSTA1*B (−567G, −69T, −52A) frequency was 0.43, whereas the alleles c.313G and c.341T of *GSTP1* were identified with frequencies of 0.28 and 0.1 respectively. The -1053T allele of the *CYP2E1* gene was observed with 0.01 frequency. We found serum α-GST concentrations in homozygous changes c.313A>G and c.341C>T of the *GSTP1* gene significantly higher at the end of anaesthesia as compared with the levels at pre-anaesthetic and 24 h post-anaesthetic time points. Moreover, *GSTA1* wild type genotype was associated with increased α-GST concentration at 24 h after the end of anaesthesia.

**Conclusions:**

*GSTP1* gene polymorphism has an impact on the perioperative serum α-GST concentration in patients undergoing sevoflurane anaesthesia. A similar association, although not statistically significant exists between *GSTA1* gene variants and perioperative serum α-GST level.

## Background

The measurement of serum glutathione S-transferase alpha (α-GST) concentration has been used in multiple studies as hepatocellular integrity or renal injury indicator in anaesthetized patients [1, 2]. It is postulated that liver injury after sevoflurane anaesthesia may occur due to reduction in the liver blood flow caused by inhaled anaesthetic or by the toxic effect of sevoflurane itself or its metabolite [3]. Particularly the fluoromethyl-2, 2-difluoro-1-[trifluoromethyl] vinyl ether, a product of sevoflurane breakdown, has been suggested as a deleterious substance [4]. Transient increase of α-GST concentration has also been reported after anaesthesia with desflurane, isoflurane, enflurane, halothane and propofol [5–7]. Glutathione S-transferases (GSTs, EC 2.5.1.18) are a superfamily of enzymes responsible for metabolism and the detoxification process of a wide range of carcinogens, toxins and drugs including anaesthetic agents by binding them with reduced glutathione [8]. Alpha GST (α-GST, GSTA1) belongs to the most essential class of GST superfamily in the human liver. It is widely used as a more sensitive biomarker of the liver function than conventionally analyzed aspartate aminotransferase (AST) and alanine aminotransferase (ALT) because of its smaller molecular weight and shorter half-life [9]. In hepatocellular impairment, the enzyme is rapidly released from hepatocytes into the bloodstream. Previous studies demonstrate that the enzyme increase may occur directly after the end of sevoflurane anaesthesia, which suggests the toxic influence of the anaesthestic, or after 24 hours, which indicates the toxic effect of metabolites.

Alpha GST is encoded by the polymorphic *GSTA1* gene (OMIM 138359). Its different genetic variants are known as altering the enzyme activity in the human liver, which may influence the individual detoxification rate and response to drugs. Therefore, *GSTA1* is a subject of intensive molecular research. Expression of *GSTA1* depends mainly on two crucial alleles GSTA1*A and GSTA1*B, containing three linked base substitutions located in promoter region: −567T> G, −69C>T, −52G>A. However, the promoter change in position −52 of the *GSTA1* gene impairs the binding process of the Sp1 transcription factor and, in effect, reduces the promoter activity of GSTA1*B by four-fold [10].

The *GSTP1* gene (OMIM 134660), which codes for the GSTP1-1 protein of the π-GST fraction, is widely expressed in most tissues, particularly in the lung, esophagus, and placenta [11]. The variable serum concentration of π-GST has been used in multiple studies as an indicator of preeclampsia, lung and breast cancer, response to chemotherapy and drug hepatotoxicity [12–14]. Three polymorphic alleles of the *GSTP1* gene, GSTP1*A, *B and *C, characterized by two amino acid substitutions p.Ile105Val (c.313A>G) and p.Ala114Val (c.341C>T) determine the enzyme’s activity [15]. Both changes, in codon 105 and 114 are responsible for the enzyme’s decreased activity [16], because of their location in the active site of the GSTP1-1 protein.

Sevoflurane, one of the safest and commonly used anaesthetic agent, is metabolized only in 5 % by cytochrome P450, family 2, subfamily E, polypeptide 1 (CYP2E1) encoded by *CYP2E1* gene (OMIM 124040), mainly in the liver. Actually 14 *CYP2E1* alleles, associated with variable enzyme activity, have been described. Two most relevant variants in the promoter region -1053C>T and -1293G>C are responsible for increased transcription of *CYP2E1* [17]. Polymorphism in this gene was intensively investigated in the context of cancer risk and response to drug treatment.

It is presumed that the background of the variability in sevoflurane biotransformation and its possible hepatotoxic effect may constitute the genetic polymorphism of genes coding for metabolizing enzymes [18, 19]. Moreover, until now, there have been no results regarding the genetic background in the safety of sevoflurane anaesthesia. Therefore, the aim of our study was to verify whether an association exists between gene variants *CYP2E1* (−1053C>T), *GSTA1* (−567T>G, −69C>T and -52G>A), *GSTP1* (c.313A>G and c.341C>T) and perioperative serum α-GST concentrations in Polish patients undergoing sevoflurane anaesthesia.

## Methods

### Patients

A group of 86 unrelated Polish patients (50 males and 36 females) representing ASA physical status I-II, undergoing laryngological surgery under general anaesthesia with sevoflurane, were enrolled in this study. Patients ranged in age from 19 to 75 years old. The body mass index averaged 26.78 (±4.67). The mean sevoflurane exposition time was 39.15 (±23.75) min. Exclusion criteria included patients with hepatic disease or abnormal hepatic disfunction, excessive alcohol consumers and cigarette smokers, patients receiving drugs affecting hepatic enzymes.

The study was approved by the local ethics committee of the University of Medical Sciences in Poznan, Poland (Resolution No. 653/09) on 18 June 2009. One day before surgery, patients were informed about the procedure, and all of them gave written consent. Midazolam premedication was administered. For induction, propofol (1 to 2 mg/kg body mass) and fentanyl (1 to 3 μg/kg body mass) were given followed by intravenous administration of vecuronium (0.1 mg/kg body mass). Then orotracheal intubation was performed. For maintenance of anaesthesia, the volatile agent sevoflurane (Sevorane; Abbott Laboratories, Abbott Park, Illinois) was used. Ventilation parameters were adjusted so that end-tidal carbon dioxide was 30 to 40 mmHg (tidal volume 7 to 10 mL/kg, and respiratory frequency 12 breaths/min). 1.5 % to 4.0 % sevoflurane was given as 1.5 minimal alveolar concentration (MAC) total fresh gas flow at 1 L/min using a vaporizer (model 19.3; Dräger Medical GmbH, Lübeck, Germany) (Low-flow Sevoflurane anesthesia). Systolic, diastolic, and mean arterial pressures, pulse rate, peripheral O_2_ saturation, and end-tidal gas values were measured before induction of anesthesia and continuously during surgery. The total dose of sevoflurane administered to each patient was calculated in MAC hours. In the termination phase of anesthesia, the vaporizer was turned off 10 to 15 min before the end of surgery, and low flow was maintained at the rate of 1 L/min. With the recovery of spontaneous ventilation, 100 % O_2_ at the rate of 5 L/min was administered before extubation.

### Samples and laboratory analysis

Blood samples for serum α-GST measurement were collected in three time points: directly before induction of anaesthesia (T_1_), at the end of anaesthesia (T_2_) and 24 h after the end of anaesthesia (T_3_). The serum was separated and stored at −20 °C. α-GST concentrations were determined with an enzyme-linked immunosorbent assay (ELISA) (GST-α ELISA Kit; Immundiagnostik, Bensheim) according to the standard assay procedure. The absorbance was measured on a plate reader at a wavelength of 450 nm (Asys UVM 340; Biochrom Ltd, Cambridge, UK). All samples from each patient were analyzed in the same assay run. α-GST concentration was quantified against a standard curve. Assays were run in duplicate and the intra-assay variability coefficient was <5 %. The reference of α-GST concentration ranged from 0.6 to 20 μg/L.

### Molecular analysis

Genomic DNA for molecular analysis was obtained from peripheral blood samples according to standard procedures using the method with guanidine isothiocyanate (GTC). Analysis of *GSTA1* promoter variants (in positions −52, −69 and −567) and two substitutions (c.313A>G and c.341C>T) in *GSTP1* gene was performed using sequencing and pyrosequencing respectively, as was described previously [20]. For genotyping of promoter SNP -1053C>T in the *CYP2E1* gene we used the pyrosequencing technique. Specific primers (forward 5′-GTGATTTGGCTGGATTGTAAATG-3′, reverse 5′-CAGACCCTCTTCCACCTTCTATGA-3′) for amplification of 239 bp-length fragment and sequencing primer (5′-AATTCATAGGTTGCAATT-3′) were designed by Pyrosequencing PSQ Assay Design Software. PCR reaction was carried out using Applied Biosystems 2720 Thermal Cycler (Applied Biosystems, Foster City, CA) on the total volume of 30 μl containing 0.9 U of FIRE Pol DNA Polymerase®, 0.2 μM of primers, 3.0 μl 10x buffer, 2.4 μl dNTP mix (2.5 mM each dNTP), 1.8 mM MgCl_2_ solution and 96 ng DNA. The program started with initial denaturation at 95 °C for 3 min, followed by 50 cycles at 95 °C for 30 s, 60 °C for 30 s, and 72 °C for 60 s. All reagents were obtained from Solis BioDyne (Tartu, Estonia). Pyrosequencing was performed by PSQ™96MA System (Qiagen) and PyroMark Gold Q96 Reagents (Qiagen GmbH, Hilden, Germany) as described by the manufacturer.

### Statistical methods

For determining the normality of distribution of serum α-GST levels, Shapiro-Wilk’s normality test was used. The repeated measures analysis of variance (ANOVA) was applied for evaluation the changes in α-GST concentrations between patients at different time points. All calculations were performed using STATISTICA 10.0 software (Stat Soft, 2014). A *p*-value of < 0.05 was considered statistically significant. The statistical method applied is in line with the experience scheme [21].

## Results

A total of 86 individuals were screened for genetic variants -52G>A (rs3957356), −69C>T (rs3957357) and -567T>G (rs4715332) in the *GSTA1* gene promoter region, two substitutions c.313A>G and c.341C>T in *GSTP1* gene and the promoter change -1053C>T in *CYP2E1* gene. The allele GSTA1*B (−52A, −69T, −567G) frequency was 0.43 (while each allele combination frequencies were 0.34 for A*/A*, 0.46 for A*/B* and 0.20 for the B*/B*). Allele c.313G of *GSTP1* gene produced frequency of 0.28 (genotype A/A were observed with 0.52 frequency, genotypes A/G and G/G with 0.38 and 0.1 frequency respectively). Whereas allele c.341T of *GSTP1* gene was detected with 0.1 frequency (genotype C/C with 0.84, C/T with 0.13 and T/T with 0.03 frequency were observed). The *CYP2E1*-1053T allele had 0.01 frequency.

**Fig. 1:**
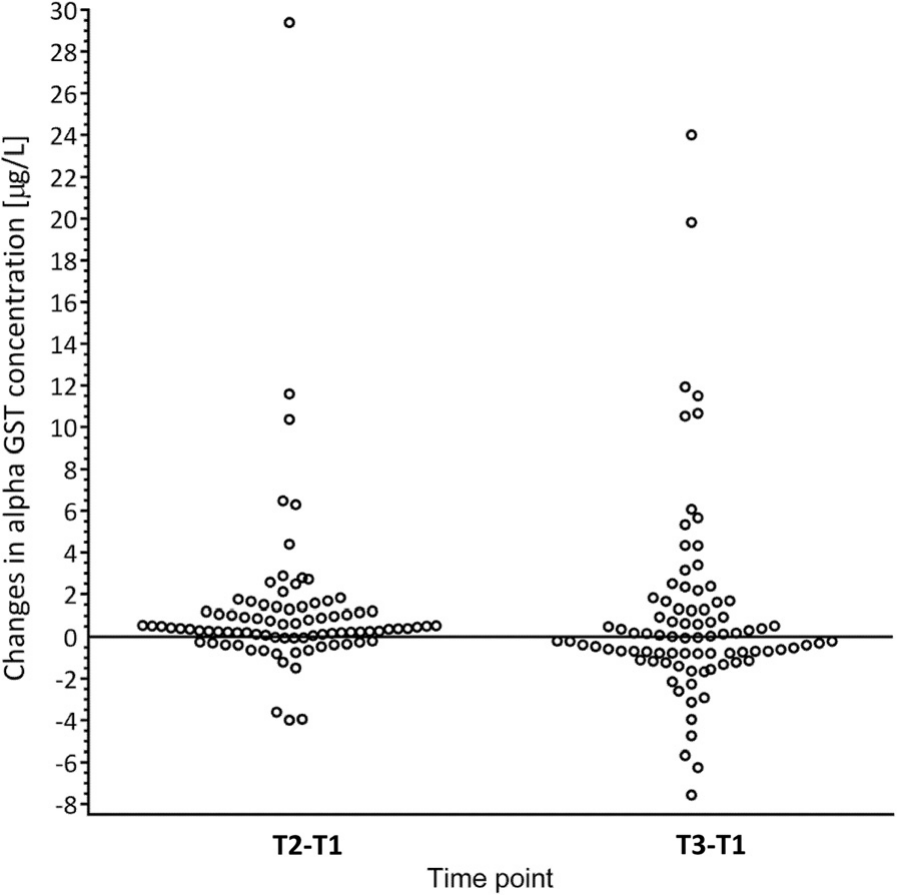
The changes in serum α-GST levels. Left plot - the differences between serum α-GST concentrations at the end of anaesthesia (T2) and before anaesthesia (T1). Right plot - the differences between serum α-GST concentrations at 24 h after anaesthesia (T3) and before anaesthesia (T1)

**Fig. 2:**
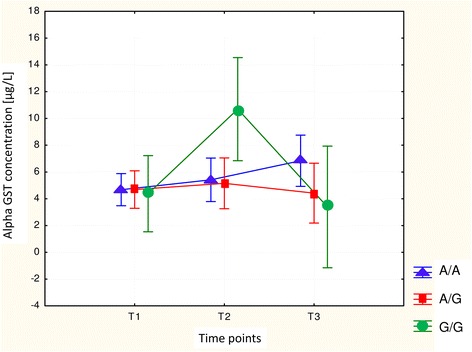
Perioperative changes in the serum α-GST level depending on genotype A/A, A/G, G/G at position 105 of the *GSTP1* gene

**Fig. 3:**
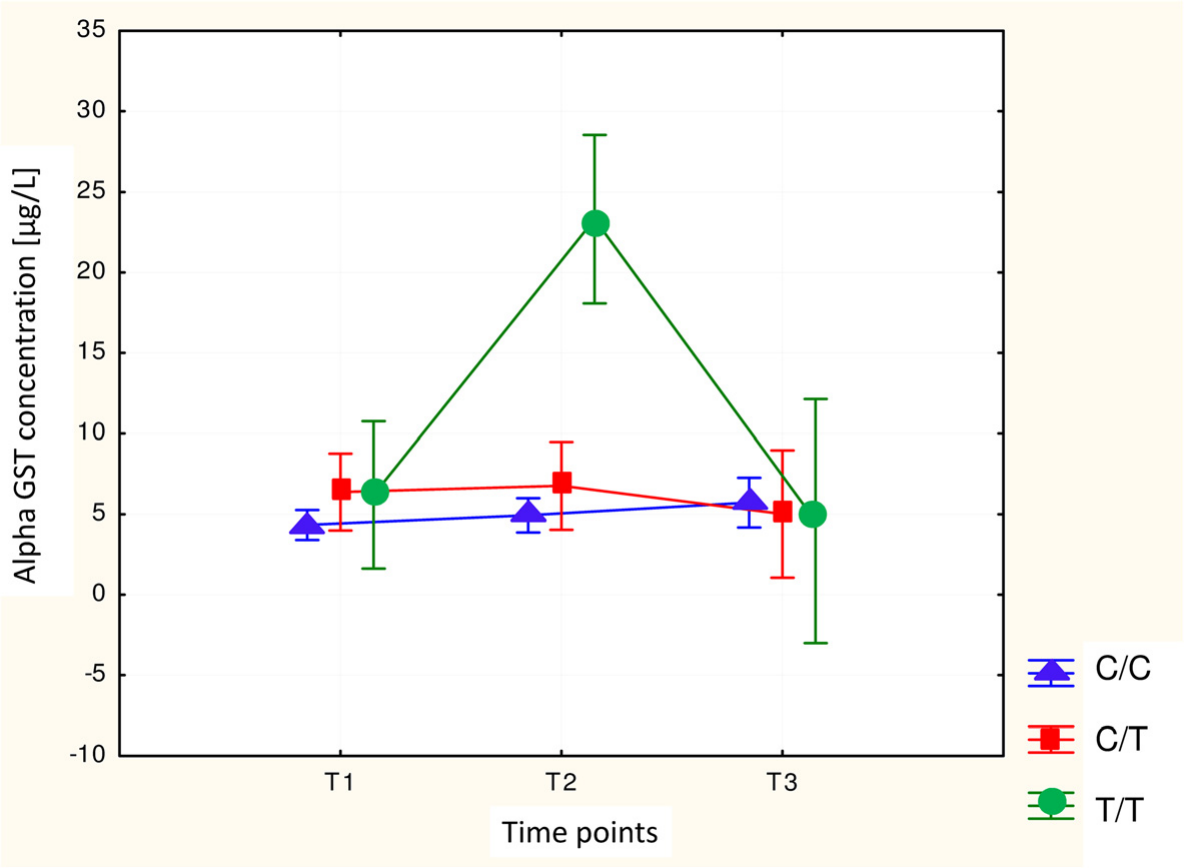
Perioperative changes in the serum α-GST level depending on genotype C/C, C/T, T/T at position 114 of the *GSTP1* gene

**Fig. 4:**
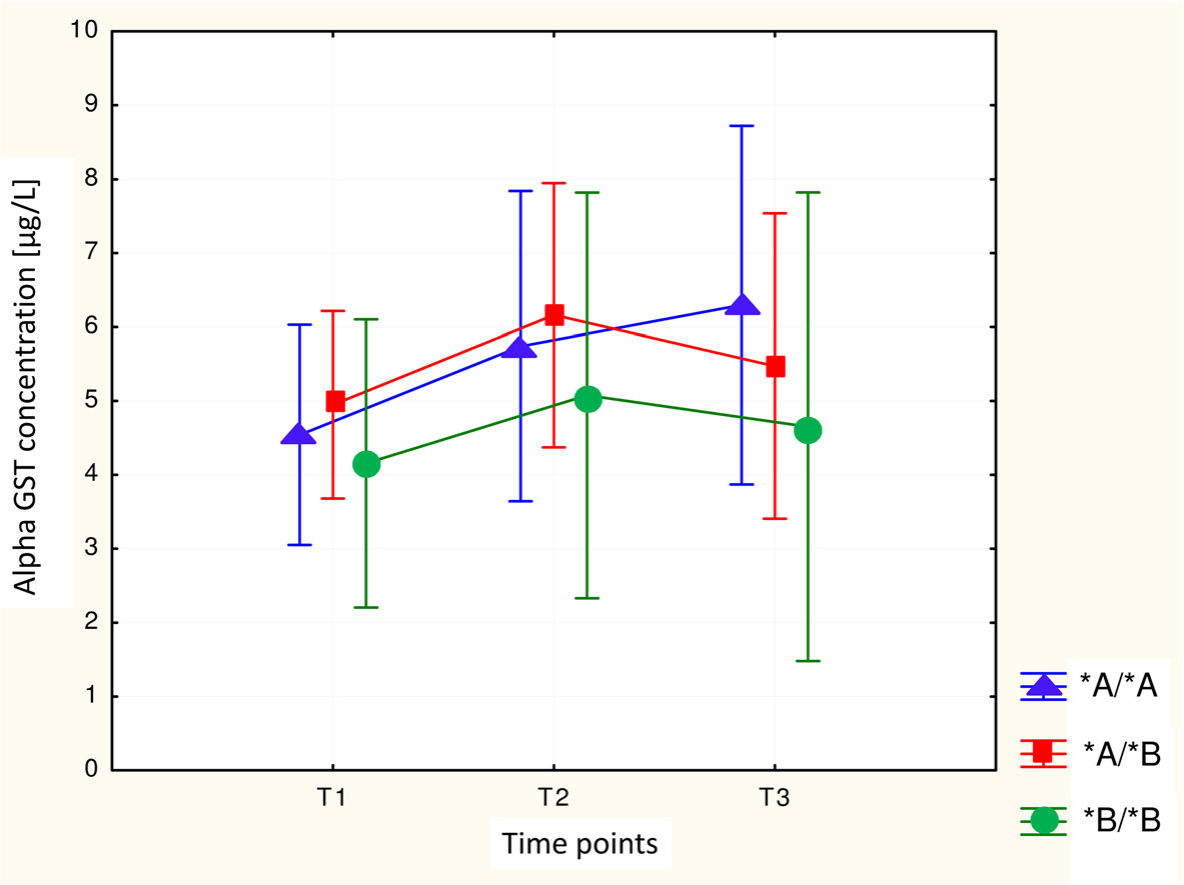
Perioperative changes in the serum α-GST level depending on alleles *A/*A, *A/*B and *B/*B of the *GSTA1* gene

In the whole study group we have measured serum α-GST concentration in three perioperative time points. We observed the individual and inter-individual differences in enzyme concentration levels. The increase in α-GST level (minimum of 20 % compared to a basal value T1) in 39.5 % of patients at the end of anaesthesia (T_2_) and in 27.9 % of subjects at 24 h after the end of anaesthesia (T_3_) was observed. The perioperative changes in α-GST concentration are illustrated in (Fig. 1). In individuals with *GSTP1* c.313G/G genotype the early postoperative serum α-GST concentration was significantly higher, compared with the preanaesthetic and 24 h postanaesthetic measurements. Changes in α-GST concentrations between patients were evaluated at different time points using repeated measures analysis of variance where *p* = 0.00004 (Fig. 2). Similarly, patients carrying *GSTP1* c.341TT genotype demonstrated significantly higher α-GST concentration when compared with basal and 24 h postanaesthetic values using the same test (*p* = 0.0000) (Fig. 3). Moreover, we found wild type homozygotes of *GSTA1* (−52C/C, −69G/G, −567A/A) as associated, but not statistically significantly, with increased α-GST concentration at 24 h after the end of anaesthesia, when compared with the preoperative and early postoperative values (Fig. 4). The promoter change -1053C>T in *CYP2E1* gene had no impact on the enzyme profile during the analyzed perioperative time in patients anaesthetized with sevoflurane.

## Discussion

Human GST was indicated in numerous studies as a sensitive marker of hepatocellular impairment [9, 22]. The enzyme is released from the hepatocytes into the bloodstream if hepatocellular integrity is disrupted. Measurements of serum α-GST concentration have been used many times in studies analyzing the influence of anaesthetics on hepatocellular integrity. Up to now, the results regarding sevolfurane have suggested mild impairment, but the background of this effect is not clear. Ray et al. [3] has demonstrated the significant increase in GST at 1 h after the end of sevoflurane infusion, compared with baseline level (before anaesthesia). He also observed that some patients exhibit an increased GST level at 24 h after the end of anaesthetic infusion. Studies conducted by Taivainen et al. [7] indicated that general anesthesia with sevoflurane and halothane administered to children resulted in the increase in α-GST concentration in the serum, which returned to normal within 24 h. Similarly, Suttner et al. [2] observed minimally affected hepatic integrity after desflurane and sevoflurane anaesthesia in elderly patients. Studies performed on a group of patients anaesthetized with sevoflurane showed that after 1 h, serum α-GST level increased in both groups [1]. Kaymak et al. [23] also has proved the early postoperative increase in serum α-GST in patients after sevoflurane anaesthesia.

Our study has also demonstrated that sevoflurane anaesthesia resulted in a transient early postoperative increase in serum α-GST concentration. Generally, the observed enzyme growth was mild, with the exception of individuals carrying *GSTP1* c.313G/G, or c.341 T/T genotype, where the enzyme rise was statistically significant when compared with preanaesthetic and 24 h postanaesthetic level. Elevated enzyme concentrations mostly returned to the baseline within 24 h. However, α-GST concentrations were still elevated at 24 h after anaesthesia in wild type homozygotes *GSTA1* -52C/C, −69G/G, −567A/A.

The first trials to correlate the variability of GST enzyme concentration with the genetic polymorphism were performed by Kaymak et al. [23]. He confirmed that *GSTP1* heterozygote c.313A/G had significantly higher serum α-GST concentrations at 24 h compared with the wild type genotype. Our study has not confirmed these results. Although we have also tested the influence of p.Ile105Val change on the enzyme level, it seemed more likely that there would be an association between polymorphism in the *GSTA1* or *CYP2E1* gene and enzyme level, because *GSTA1* gene is responsible for synthesis of the α-GST protein, and *CYP2E1* gene codes the main enzyme involved in sevoflurane biotransformation.

In our study, all selected polymorphisms cause altered enzyme activity: *CYP2E1* (−1053C>T) increases it, and *GSTA1* (−567T>G, −69C>T, −52G>A) and *GSTP1* (c.313A>G, c.341C>T) changes reduce it [10, 16, 17]. These alleles are described in the literature as strongly associated with the variable efficiency of metabolism and detoxification of numerous compounds, as well as with drug therapy and susceptibility to diseases. Any changes in the sevoflurane biotransformation rate (both in metabolism and in the detoxification process) may pose a risk of hepatotoxic effect of sevoflurane [18].

The increase in α-GST concentration at the early postoperative time point may be explained by the evidence suggesting that sevoflurane reduces total hepatic blood flow in humans [24]. There is no clear suggestion of a hepatotoxic metabolite of sevoflurane, which might initiate the damage of hepatocytes.

In our study two polymorphisms in *GSTP1* gene (p.I105V and p.A114V) proved to be relevant to the transient hepatocellular impairment in patients after sevoflurane anaesthesia. Although the polymorphism at codon 105 of *GSTP1* gene was also found to be crucial in the study by Kaymak et al. [23], the effect on the change in enzyme level is different in our study.

## Conclusions

The present study is a preliminary work and further analyses on larger populations and longer infusion times are needed. However, in summary, we can conclude that *GSTP1* p.I105V and p.A114V variants have an impact on the increased serum α-GST concentration in patients undergoing sevoflurane anaesthesia. A similar association, although not statistically significant exists between *GSTA1* gene variants and perioperative serum α-GST level.

## Abbreviations

α-GST: Serum glutathione S-transferase alpha
ANOVA: Analysis of variance
ALT: Alanine aminotransferase
AST: Aspartate aminotransferase
CYP2E1: Cytochrome P450, family 2, subfamily E, polypeptide 1
ELISA: Enzyme-linked immunosorbent assay
MAC: Minimal alveolar concentration
